# Distinct motors, shared mechanics: unifying principles of microbial gliding

**DOI:** 10.1128/jb.00050-26

**Published:** 2026-04-01

**Authors:** Samyabrata Sen, E. C. Henderson, Ferran Garcia-Pichel, Mohammed Kaplan, Abhishek Shrivastava

**Affiliations:** 1 Center for Fundamental and Applied Microbiomics, Biodesign Institute, Arizona State University 43363, Tempe, Arizona, USA; 2 School of Life Sciences, Arizona State University 69991, Tempe, Arizona, USA; 3 Center for Biological Physics, Arizona State University 7864, Tempe, Arizona, USA; 4 Department of Microbiology, University of Chicago 456539, Chicago, Illinois, USA; Dartmouth College Geisel School of Medicine, Hanover, New Hampshire, USA

**Keywords:** gliding motility, cellular motility, surface motility and colonization, host colonization and egress, *Flavobacterium *gliding, Myxobacteria gliding, *Mycoplasma *gliding, Cyanobacterial gliding, apicomplexan, *Plasmodium *or *Toxoplasma *gliding, diatom gliding

## Abstract

Gliding motility is one of the more elegant tricks in the microbial playbook, allowing cells to move smoothly along an external surface. Unlike swimming in bulk fluid, gliding requires contact with a biotic or abiotic surface, imposing strong physical constraints on how forces are generated, transmitted, and dissipated. It is an active, energy-dependent process that operates without flagella, instead relying on specialized molecular machines that couple dynamic surface adhesins to motion. In this mini-review, we summarize recent advances that illuminate how distinct molecular motors converge on common mechanical principles to drive microbial gliding motility.

## WHAT IS MICROBIAL GLIDING MOTILITY?

Gliding motility is a widespread form of microbial locomotion traditionally defined as smooth movement of a cell along an external surface, enabling active translocation that is distinct from diffusive motion (1–4). This mode of motility differs fundamentally from the gliding of macroscopic organisms, such as birds or humans using hang-gliders, which rely on inertial forces. In the microscopic world, where Reynolds numbers are low and inertia is negligible, microbes cannot coast and must continuously expend energy to move (5). Thus, in accordance with the laws of physics, gliding microbes must expend cellular energy, rely on active molecular motors to achieve smooth, continuous movement, and effectively transmit force from the molecular motors to the extracellular surface (6).

**Fig 1:**
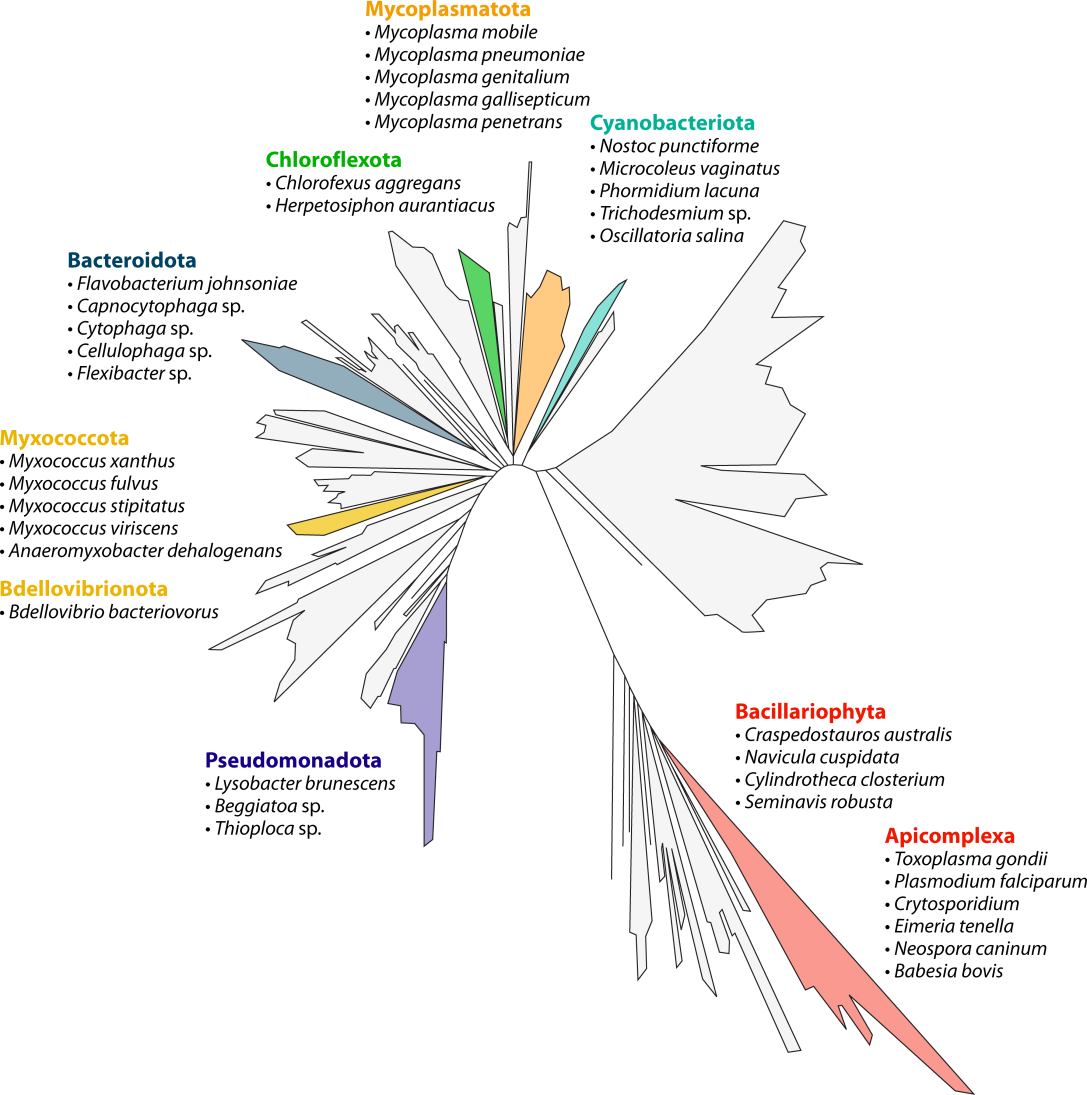
The broad diversity of gliding organisms across the tree of life shows that this form of motility has evolved independently in multiple distinct phyla. Commonalities in the physical mechanisms of gliding across the tree of life, such as the use of surface adhesins and coordinated machinery for movement along solid substrates, point to similar mechanisms across lineages. Examples of organisms in which gliding has been reported are listed for each phylum.

To glide, some component of the cell surface or a specialized appendage must transiently grab onto the external substratum. Often, though not always, this component is primed with exopolysaccharides (EPS) (3). The adhesive interaction allows outward transmission of internally generated forces to attain cellular motion with respect to the external surface. As any avid reader of the microbiology literature might imagine, this extra requirement quickly turns gliding into a mechanical juggling act, engaging many moving parts within the cell. The puzzle deepens as one considers that microbial gliding has evolved independently multiple times across the tree of life (Fig. 1), with recent molecular discoveries revealing that different branches have arrived at strikingly different molecular motors to solve the same problem of surface-associated locomotion.

Before diving into the diversity of gliding motors, it is worth clarifying where gliding ends and twitching (another distinct mode of microbial surface translocation) begins, as the boundary between these two surface-associated modes of microbial movement is not always clear, especially in literature related to cyanobacteria. The Type IV pili-driven twitching motility of single prokaryotic or archaeal cells is well-differentiated from gliding as it is characterized by jerky or snappy cell movements, rather than smooth translocation (7, 8). For this reason, common usage in bacteriology typically does not consider Type IV pili-based motility a form of gliding. A notable exception, however, is cyanobacteria, whose Type IV pilus-driven surface motility is commonly referred to as gliding because the coordinated action of multiple pili produces smooth, continuous movement. This is true for filamentous forms, though locomotion of single-celled cyanobacteria can sometimes be jerky. From a phenotypic perspective, these organisms clearly exhibit smooth surface translocation, and from a mechanical perspective, they employ a molecular motor that drives the movement of an adhesin, namely, the Type IV pilus. By these criteria, filamentous cyanobacteria satisfy the defining requirements of gliding motility. Accordingly, in this review, we follow the prevailing literature and phenotypic distinctions and consider all microbes, including filamentous cyanobacteria, that move smoothly along surfaces without the aid of flagella, as gliders.

## DIVERSE MOLECULAR MACHINERIES ENABLE GLIDING MOTILITY

**Fig 2:**
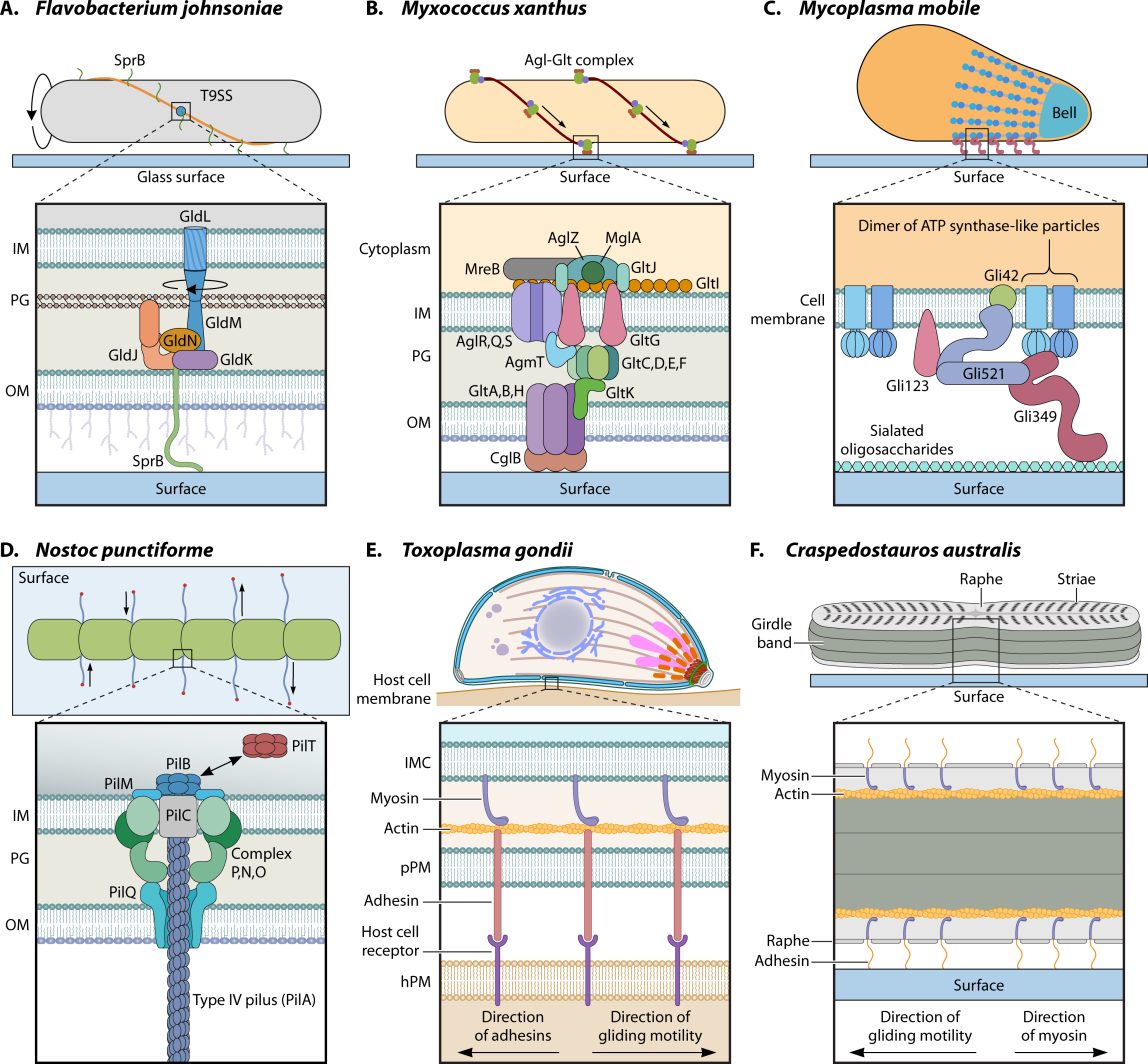
The diverse molecular mechanisms of cellular motility. (**A**) The rotary T9SS and conveyor belt of *Flavobacterium johnsoniae*. (**B**) The focal adhesin complex of *Myxococcus xanthus*. (**C**) The leg-like attachment machinery of *Mycoplasma mobile*. Additional mechanistic models of motility for *Mycoplasma genitalium* (21) and *Mycoplasma pneumoniae* (22–26) are described in the text, with additional details available in the cited literature. (**D**) Multiple Type IV pili strategically arranged at both sides of the septal wall ensure smooth, coordinated gliding in filamentous cyanobacteria. The molecular organization depicted is based on well-characterized Type IV pilus systems of cyanobacteria. (**E**) The actin and myosin-based glidosome of the apicomplexan *Toxoplasma gondii*. (**F**) The proposed raphe and actinomyosin system of the diatom *Craspedostauros australis*. (OM, outer membrane; IM, inner membrane; PG, peptidoglycan; IMC, inner membrane complex; pPM, parasite plasma membrane; hPM, host plasma membrane).

Gliding is observed across the tree of life, occurring in diverse bacterial phyla as well as in eukaryotes such as diatoms and apicomplexan parasites (Fig. 1). Well-studied bacterial model systems include members of Bacteroidota (e.g., *Flavobacterium*, *Cytophaga*, *Capnocytophaga*, *Cellulophaga* [9], and *Flexibacter* [10]), the Myxococcota (3), the Mycoplasmatota (11), and many filamentous cyanobacteria (12). Eukaryotic gliders include single-celled algae such as diatoms (*Bacillariophyta*) and parasitic apicomplexans including *Plasmodium* (13), *Toxoplasma* (14), and *Cryptosporidium* (15). Beyond these canonical systems, gliding has also been reported in additional bacterial lineages, including filamentous Chloroflexota such as *Chloroflexus aggregans* (16) and *Herpetosiphon aurantiacus* (17), as well as select members of Pseudomonadota like *Lysobacter brunescens* (18), *Beggiatoa* sp. (19), and *Thioploca* sp. (20). Key biomechanical features of representative gliding systems are summarized in Fig. 2 and discussed below.

## BACTERIAL GLIDING MOTILITY

### (A) Bacteroidota

Within the phylum Bacteroidota, gliding is widespread, whereas flagellar motility is largely absent. Most classes display robust gliding behavior as indicated by both phenotypic observations and by the presence of conserved gliding-related genes. An exception is within the Bacteroidia class, where genes required for gliding are in a subset of species, even though the Type IX Secretion System (T9SS) is more broadly conserved, suggesting a decoupling of secretion and motility (27). The primary genetically tractable model for studying Bacteroidota gliding is *Flavobacterium johnsoniae* (Fig. 2A), a rod-shaped bacterium that moves by translocating cell-surface adhesins along the length of the cell and around its poles (28, 29).

Three-dimensional tracking of both adhesins and cell bodies has revealed that adhesin motion follows a right-handed spiral trajectory, propelling the cell forward in a screw-like manner against the surface (30). The best-characterized adhesin is SprB, an ~6,500 amino acid protein that forms a ~150 nm long filament on the cell surface (28). Individual cells often express multiple types of adhesins, likely enabling efficient attachment to a variety of surfaces (31). These adhesins move along closed, pole-to-pole tracks embedded in the outer membrane in a conveyor belt-like architecture (31, 32). Cryo-electron tomography (cryo-ET) studies identified multiple such conveyor belts that are proposed to contain the proteins GldJ, GldK, and GldN (33, 34).

The gliding motility of Bacteroidota is powered by the T9SS, a proton motive force-driven rotary machinery composed of approximately 20 different proteins (35, 36). The T9SS is a bifunctional system: it not only drives gliding but also secretes various proteins, including enzymes and mobile cell-surface adhesins (37). Its rotation is powered by the GldLM stator units, which have a 5:2 structural arrangement resembling, but not homologous to, the MotAB stator units of the bacterial flagellar motor (38). Cryo-ET suggests that GldM (PorM) interacts with the GldKN (PorKN) in different conformations (35), and it is proposed that the GldLM stator units harness proton motive force to rotate and push against the outer membrane-associated GldJKN conveyor belt of the T9SS (33, 39); thus, the rotary T9SS interacts with a conveyor belt, analogous to a molecular snowmobile (6, 32, 39, 40). However, several key mysteries remain. For example, (i) are the conveyor belts anchored to the peptidoglycan layer, (ii) how do the T9SS rotors mechanically couple to these belts, and (iii) how does the cell decide which way to go? Intriguingly, deleting the C-terminal region of the conveyor-belt protein GldJ flips the rotational direction of the T9SS motor (39). Yet the identity of this signal, how it is generated, and how it is relayed to the motors are still unknown. It also remains an open question whether additional regulatory circuits, perhaps involving two-component systems, help steer this microscopic snowmobile.

### (B) Myxococcota

Gliding motility is a common feature of these soil-dwelling bacteria and is fundamental to their complex social behaviors. Myxobacteria possess two distinct motility systems. Type IV pili drive social (S) motility, which is smooth at the level of cell groups that remain in contact with one another, but leads to twitching in individual cells (41). In contrast, the gliding apparatus (adventurous or A-motility) of *Myxococcus xanthus*, the primary model organism for the Myxococcota (Fig. 2B), enables smooth translocation of single cells. This system is powered by a proton-conducting motor composed of the AglRQS proteins, part of a multiprotein complex spanning the cell envelope and structurally similar to the MotAB stators of the bacterial flagellar motor, the GldLM stators of the Bacteroidota T9SS, and the TolQR proteins (42). Related Myxobacteria like *Myxococcus fulvus* (43), *Myxococcus stipitatus* (44), *Myxococcus virescens* (45), and *Anaeromyxobacter dehalogenans* (46) are also thought to exhibit gliding motility (3, 45) through similar mechanisms. Agl proteins are predicted to function as ion-driven stator-like motors, likely sharing the 5:2 architecture described for MotAB/TolQR-class systems, although their structure has not been solved and direct evidence of rotation is lacking (42, 47). Interestingly, unlike the fixed stators of the flagellar motor and T9SS machinery, Agl proteins are thought to travel along helical cytoplasmic tracks (48, 49).

The Agl machinery also depends on the bacterial actin homolog MreB, since its disruption impairs the helical motion of the motors. The interaction between Agl motors and MreB organizes the gliding machinery into a cytoplasmic platform that supports coordinated movement (50–52). The transmembrane protein AglQ links to MreB via the large scaffold protein GltI and its partner GltG, which together form part of the intracellular scaffold that couples Agl motors (53) to the MreB-associated cytoskeletal track (47, 54). The Agl motors also connect with a trans-envelope Glt complex (including Glt A, B, C, D, E, F, H, J, and K) that spans the periplasm and outer membrane where it assembles bacterial focal adhesins to transmit force externally. The Glt complex recruits and retains the surface protein CglB, which contains a von Willebrand domain and mediates strong substrate binding. CglB-loaded complexes are proposed to form focal adhesions whose helical motion drives screw-like gliding of the cell (47, 55). Additionally, the lytic transglycosylase AgmT may couple the gliding motors to the peptidoglycan layer by modifying it to anchor focal adhesins, thereby enabling efficient force transmission (56). How Agl motors that travel along the cell length transmit force to proteins embedded in the peptidoglycan remains unknown, and it is also unclear whether these motors rotate like the stator units of the bacterial flagellar motor. If Agl motors do rotate, it remains to be determined whether their rotation is mechanically coupled to peptidoglycan-anchored components during focal adhesion formation. Structural studies of the gliding machinery using cryo-ET, together with single-motor assays that have been highly informative for other molecular motors, could help resolve these outstanding questions about the mechanism of myxobacterial gliding.

### (C) Bdellovibrionota

*Bdellovibrio bacteriovorus*, a member of the phylum Bdellovibrionota, is a predatory bacterium that invades the periplasm of other gram-negative bacteria (57). While prey searching relies on rapid swimming (up to 160 µm s^−1^) driven by a single polar sheathed flagellum (58–61), a slower form of gliding motility (15–20 µm h^−1^) is used to move along either abiotic or prey surfaces. This gliding is independent of both flagella and pili and is essential for prey invasion and progeny release (62). Recent studies implicate the second messenger 3′,3′-cGAMP in regulating gliding behavior (63), but the underlying motility machinery remains unidentified. Genomic analyses reveal partial conservation of myxobacterial gliding components, including homologs of *aglQ*, *mglA*, and *romR*, alongside notable absences or divergences (62, 64–66). These observations suggest a distinct, only partially conserved gliding system. Identifying the motor, adhesins, and force-transmission mechanisms in *Bdellovibrio* represents a major open challenge with implications for understanding bacterial predation.

### (D) Mycoplasmatota (Mycoplasma)

Several species of *Mycoplasma* exhibit a unique form of gliding motility despite lacking cell walls, flagella, and pili (67, 68). These organisms possess reduced genomes (500–700 genes) and have likely evolved gliding as an adaptation for host colonization and immune evasion (69, 70). The fastest known gliding Mycoplasma species is *Mycoplasma mobile* (Fig. 2C), which glides with the speed of 2–5 µm s^−1^ utilizing adhesins that bind to sialylated oligosaccharides present on host cells. It is proposed that the whole system is akin to a combination of adhesins with a rotary ATPase (71). Multiple proteins assemble to form the gliding apparatus in *M. mobile*, which can be structurally divided into two distinct parts: internal and surface exposed (72–75). Cryo-ET (76) and recent negative staining electron tomography work have revealed its structural features (73, 74). The internal part, located in the cytoplasm, is comprised of a 440 nm wide, bowl-shaped bell structure adorned with a honeycomb structure at its surface. Attached to this bell are 46 chains, each 430 nm long, containing 17 distinct particles each that are reminiscent of the subunits of ATP synthase. The surface-exposed part contains proteins like Gli349, Gli521, and Gli42, which act as receptors for the host’s sialylated oligosaccharides and function as a crank for force transmission, acting like tiny legs to move the cell in centipede-like fashion (73).

In contrast, *Mycoplasma pneumoniae* glides slowly (~0.5 µm s⁻¹) using an inchworm-like mechanism (67, 68, 77) driven by a specialized terminal attachment organelle that contains complex cytoskeletal elements resolved by cryo-ET (22–26, 34, 68, 71, 78–80). Similarly, *Mycoplasma genitalium* moves using a terminal organelle through a mechanism involving spring-like compression and ratcheting (21, 81–83). Attachment organelle-driven motility has also been reported in *Mycoplasma gallisepticum* (84) and *Mycoplasma penetrans* (85). Despite their mechanistic diversity, all Mycoplasma gliding systems rely on ATP-driven internal machinery mechanically coupled to surface adhesins, illustrating a shared physical strategy implemented through distinct molecular architectures. Further comparative analyses across gliding and non-gliding species might help determine whether these systems derive from a conserved ancestral module or represent independent adaptations for host-associated surface motility.

### (E) Cyanobacteria

While a large proportion of cyanobacterial species are motile, none possess flagella. With the exception of some strains of marine unicellular *Synechococcus*, which display a mysterious form of swimming (86), all other motile members of the Phylum move by engaging the Type IV pilus (87), either against a surface or against other cyanobacteria. In filamentous cyanobacteria, however, this type of movement is traditionally referred to as gliding motility, primarily because, as opposed to twitching, cyanobacterial Type IV pilus causes smooth motion of the cell, possibly due to multiple pili working in a synchronized manner as shown in Fig. 2D (88). In the unicellular *Synechocystis*, however, locomotion can appear twitchy and is called twitching in single cells, but it can also be smooth in populations of cells (89, 90). Additionally, cyanobacterial motility is invariably associated with the excretion of exopolysaccharides (EPS) that are deposited on the substratum and left behind as trails.

Several mechanistic models for the generation of propulsion in gliding cyanobacteria were brought forward during the last half century, including the contraction waves of continuous cell surface fibrils along trichomes in filamentous forms (a sort of reptation) (91) and the directional excretion of polysaccharide through rings of junctional pore rings (in a type of jet propulsion) (92). However, direct evidence for either one of these mechanisms is still lacking. Gliding motility has been reported and studied phenomenologically in several cyanobacteria, including *Microcoleus vaginatus* (93), *Trichodesmium* sp., and *Oscillatoria salina* (94). More recently, genetic evidence in three different cyanobacteria of widely divergent phylogeny, the unicellular *Synechocystis* PCC6803, the simple filamentous *Phormidium lacuna* HE10DO, and the complex heterocystous filamentous *Nostoc punctiforme* PCC73102, in which developmentally specialized short filaments known as hormogonia are the only motile phase in its life cycle, clearly pointed to the direct involvement of Type IV pili (95). In all three cases, knock-out of one or more of the Type IV pili genes resulted in non-motile phenotypes. Hence, Type IV pili-based motors are currently regarded as the most common motility mechanism throughout the phylum (96–99). Pili are polymerized and extend outside of the cell, adhering to the substrate at the distal end. The subsequent depolymerization-driven pilus recoiling pulls the cell in the direction of the surface attachment point.

Several key proteins are involved in the process: the major pilin monomer PilA; the motor ATPase PilB, which catalyzes pilus polymerization and extension in cooperation with the inner membrane protein PilC; and a second motor ATPase (PilT) that drives pilus retraction in coordination with PilC. Secretins PilQ/GspD may also be involved as a gateway for extrusion of pili. Type IV pilus-driven movement, often observed as a jerky form of surface-dependent motility, is commonly observed in bacteria from various phyla, including *Pseudomonas*, *Neisseria*, and *Myxococcus* (8), where it is usually referred to as twitching motility.

Notable differences have been observed in how unicellular and filamentous cyanobacteria achieve directionality. In *Synechocystis*, this is enabled by localizing PilB on the leading end of the cell, promoting preferential pili extension there (100, 101), as is common in other twitching unicellular bacteria (102), while in *Nostoc* hormogonia, pili localize preferentially in rings around the cell junctions, on both sides of the septum midpoint, facing opposite directions. Directionality here is thought to be attained by the coordinated pilus extension on rings facing the same direction at multiple cell junctions along the filament, switching to the opposite side when direction reversals are required (98). The genetic locus *hmp*, whose chemotaxis-like proteins localize around these septal rings, seems to be directly involved in facilitating the switch (103). The secretion of polysaccharides is also intimately associated with gliding in cyanobacteria at the molecular level, being, in fact, indispensable for it. Knockouts of genes coding for enzymatic components involved in the synthesis (Hps) and extrusion (CrtB) of EPS result in a loss of gliding in *Nostoc* hormogonia (98, 104, 105). The role of EPS is currently regarded as important in conditioning the surface for effective gliding (106), as not all types of surfaces can support gliding equally or at all (107). A uniform layer of EPS that adheres to the substratum but not to the cell surface can reduce overall friction and homogenize spatial variability for a smoother ride. That the production of EPS in gliding is so much more conspicuous and tightly imbricated with the Type IV pili motors in filamentous cyanobacteria than in other bacteria may have to do with the much larger viscous drag associated with moving cells that are generally much more massive (108) than those of typically sized bacteria. Consistently, gliding speeds attained by *Nostoc* correlate positively with the amount of polysaccharide excreted (109).

## EUKARYOTIC GLIDING MOTILITY

### (A) Apicomplexa

Gliding motility is not an adaptation solely reserved for bacteria. Comparable surface-associated movement has independently evolved in several eukaryotes, most notably parasitic apicomplexans such as *Plasmodium* (the causative agent of malaria), *Toxoplasma gondii* (the causative agent of toxoplasmosis), and *Cryptosporidium* (the causative agent of cryptosporidiosis). Motility enables them to navigate along host tissues and invade host cells, an essential feature for their pathogenicity.

Gliding motility in Apicomplexa (Fig. 2E) relies on the “glideosome” which is a dynamic layer of filamentous actin (110–113), a fast, single-headed myosin (MyoA), and several gliding-associated proteins (114–117). The glideosome is primarily confined between the plasma membrane and an underlying membranous scaffold called the inner membrane complex. The prevailing mechanistic model suggests that myosin exerts force on short actin filaments, forcing them to slide to the posterior end of the cell through the intermembrane space, thus producing a rearward directional force. Actin-linked adhesin proteins on the parasite’s outer surface bind to a stationary external substrate (such as a host cell surface); this attachment, paired with internal myosin force, results in forward parasitic gliding motility (118, 119). Similar motility machineries have also been seen in other apicomplexans such as *Plasmodium falciparum* (120), *Cryptosporidium parvum* (15), *Eimeria tenella* (121), *Neospora caninum* (122), and *Babesia bovis* (123).

Far from being rigid tracks, actin filament networks in *Toxoplasma gondii* behave like a continuously flowing material, constrained only by the cell’s shape and size (111). This fluidity supports a surprising repertoire of self-organized motions where *Toxoplasma* or *Plasmodium* parasites can glide in helices, trace circular paths, or even spin upright in a maneuver known as twirling, where the posterior end stays anchored to the surface. In the three-dimensional, matrix-rich environment of host tissues, these same motions can morph into corkscrew-like trajectories, allowing parasites to bore their way forward while maintaining surface contact (124, 125).

Although high-resolution structures of the apical platforms that interface with the gliding machinery have recently been resolved (126), many questions remain about how the different components of the gliding machinery are coordinated in space and time and how their combined forces generate the diverse modes of parasite movement that may aid tissue penetration or immune evasion. Looking ahead, direct measurements of force production, actin turnover, and adhesion dynamics in living cells could help link molecular activity to whole-cell trajectories. Comparative studies with bacterial gliding systems may also reveal shared “rules of motion” that could enable new strategies to inhibit motility and block infection

### (B) Bacillariophyta (Diatoms)

Diatoms are single-celled algae enclosed in a rigid, silica-based shell with intricate nanoscale architecture. Within this group, raphid diatoms have evolved a specialized longitudinal slit called the raphe, which serves as a conduit for gliding along surfaces. Across species, diatoms display a striking range of gliding trajectories, including straight runs, gentle arcs, and closed circular paths (127). Diatoms colonize a wide array of environmental surfaces in lakes and oceans where they play an important role in global carbon storage and nutrient cycling (128, 129). It was recently reported that ice-dwelling diatoms, which potentially contribute to ice crystallization and carbon cycling, are not only capable of gliding across ice surfaces but exhibit enhanced motility even at subzero temperatures (130). How diatoms generate gliding motion is still not resolved, but leading models invoke an actomyosin-based motility system operating beneath the silica shell (131). A key insight comes from bead-tracking experiments in which particles attached to the raphe move back and forth at speeds that closely match those of a gliding cell. This coordinated bead motion points to molecular motors (potentially similar to those of apicomplexans) linked through a continuous biomolecular network that provides both adhesion and traction against the substrate (121). Intracellular vesicles are thought to secrete mucilage into the raphe, producing extracellular strands that tether the cell to its surroundings. Actin cables, coupled to myosin through transmembrane connectors, pull on these mucilage anchors, transmitting force to the substrate and driving cell movement in the direction opposite to myosin activity (131). The motion, however, is not always smooth. Some beads move in short, discontinuous bursts, suggesting elastic snap-like events embedded within the system. While much still needs to be determined on a molecular level, these observations suggest that diatom gliding may combine steady motor-driven transport with elastic elements that intermittently store and release mechanical energy, adding an additional layer of complexity to the mechanism (132). A schematic representation of this underlying mechanism is shown in Fig. 2F for *Craspedostauros australis* (133). Similar raphe-based motility mechanisms have been reported in other diatoms such as *Navicula cuspidata* (134), *Cylindrotheca closterium* (135), and *Seminavis robusta* (136). However, many pieces of the diatom motility puzzle are still missing. The identity and organization of the motors beneath the silica shell, how actin–myosin forces are transmitted through the raphe, and how mucilage secretion is coordinated with movement all remain open questions. While it was recently reported that dynamic switching of contact sites between the cell and the external substratum modulates path curvature (137), the molecular controllers of directionality remain unknown. Bridging live-cell imaging, molecular genetics, and biophysical measurements could reveal how these silica-armored cells manage to glide so gracefully across solid surfaces.

## CONCLUSION

As this review makes clear, microbial gliding underpins many pivotal biological processes. Yet, in most systems, we are only beginning to understand how the underlying molecular engines operate. With rapid advances in molecular tools, structural biology, rapid genotyping, and the exploration of microbial diversity, the field now stands at an exciting threshold. The coming decades promise not only to resolve long-standing mechanistic questions but also to uncover entirely new strategies by which cells convert cellular energy into directed movement over surfaces. In this light, microbial gliding offers a landscape rich in open problems, bringing together physics, evolution, and biology and inviting fresh ideas that will reshape our understanding of cellular signaling, dynamics, collective behavior, community organization, and bioenergetics across the tree of life.
