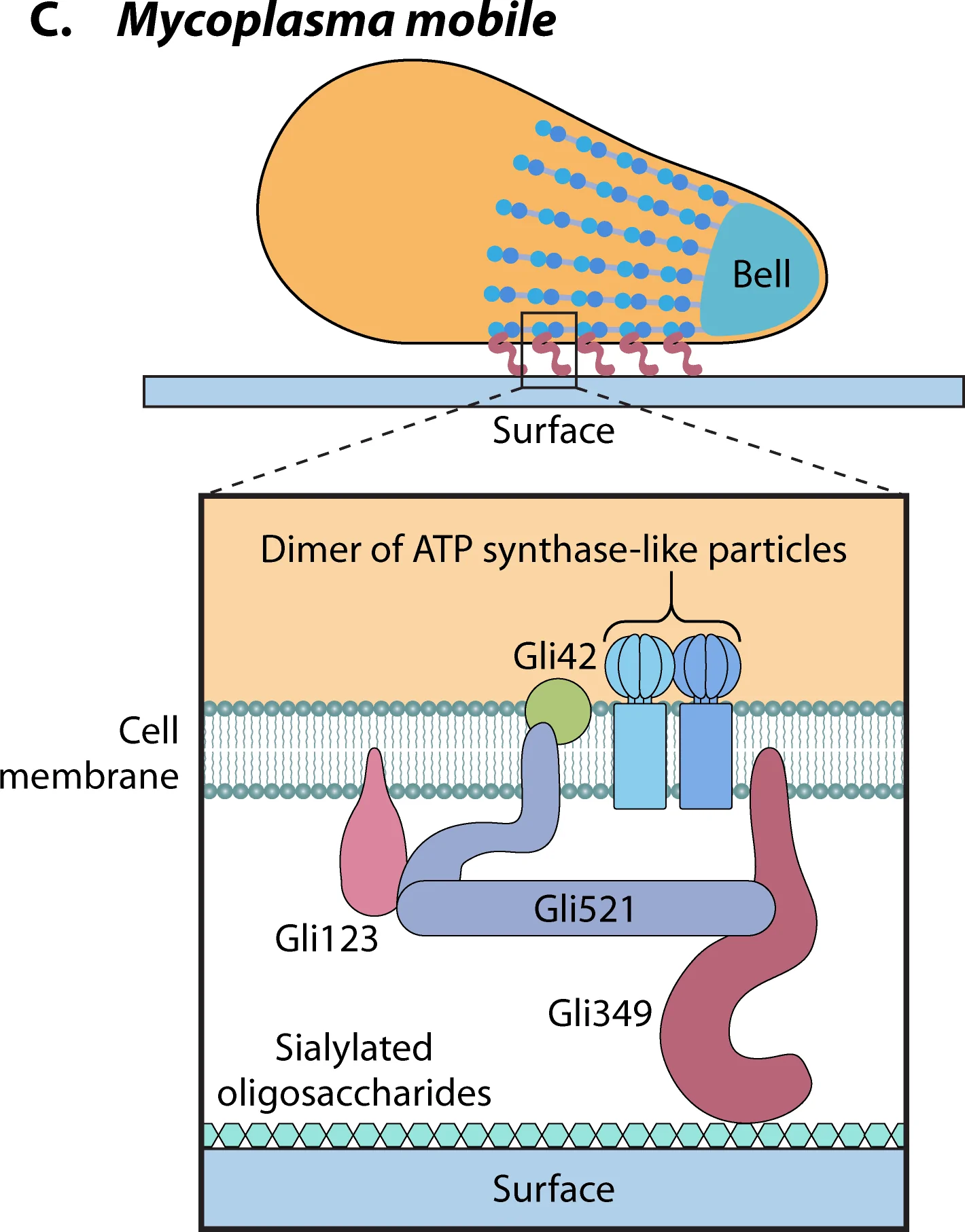# Correction for Sen et al., “Distinct motors, shared mechanics: unifying principles of microbial gliding”

**DOI:** 10.1128/jb.00215-26

**Published:** 2026-08-25

**Authors:** Samyabrata Sen, E. C. Henderson, Ferran Garcia-Pichel, Mohammed Kaplan, Abhishek Shrivastava

## AUTHOR CORRECTION

Volume 208, no. 4, e00050-26, 2026, https://doi.org/10.1128/jb.00050-26. The orientation of ATP synthase in Fig. 2C was inadvertently inverted. Figure 2C should appear as shown in this Author Correction.

**Fig 2:**